# Health Insurance Coverage for Asymptomatic Seborrheic Keratoses Treatment

**DOI:** 10.7759/cureus.42932

**Published:** 2023-08-04

**Authors:** Eseosa Fernandes, Harry Dao

**Affiliations:** 1 Preventive Medicine, University of Maryland School of Medicine, Baltimore, USA; 2 Dermatology, Loma Linda University Health, Loma Linda, USA

**Keywords:** ethics and professionalism, medical insurance, cosmetic dermatology, health equity, seborrheic keratosis

## Abstract

Seborrheic keratosis is one of the most common benign cutaneous neoplasms encountered in dermatology practice. However, treatment of asymptomatic seborrheic keratosis is not covered by health insurance. Patients frequently report being bothered by the lesions but decline treatment to avoid incurring “out of pocket” expenses. In this commentary, the authors explore the various conundrums associated with this and the implications for health equity and ethical medical financing.

## Editorial

Seborrheic keratosis is one of the most common benign cutaneous neoplasms encountered in dermatology practice. It is estimated to affect approximately 30% of the adult population, with a higher prevalence and higher number of lesions seen with increasing age [1]. Findings from a cross-sectional survey showed that dermatologists in the United States diagnose seborrheic keratoses in approximately 155 patients monthly, and 33% of these patients have more than 15 lesions [2]. The diagnosis of seborrheic keratosis is typically made based on clinical examination, which can be aided by dermoscopy. In cases where the clinical diagnosis is uncertain, a biopsy may be helpful to distinguish it from other benign and malignant proliferations [3]. Treatment of seborrheic keratoses includes the use of tangential shave removal, cryotherapy, curettage and/or electrocautery, ablative laser surgery, or a focal chemical peel.

As seborrheic keratoses are benign, treatment is not mandatory. Most private insurance companies, Medicaid and Medicare, do not pay for the removal of seborrheic keratoses if done only for cosmetic reasons. Covered medical reasons for treatment include the lesion having one or more of the following characteristics: symptomatic with bleeding, intense itching, inflammation (e.g., pain, edema, or erythema), or exhibiting sudden growth, purulent drainage, or ulceration. In addition, obstruction of an orifice, interference with vision, becoming traumatized, and/or needing to be evaluated to rule out skin cancer are reasons for a biopsy or removal of these lesions as well.

A cross-sectional study that examined patients’ perspectives regarding treatment for asymptomatic seborrheic keratoses showed that many patients are interested in treatment and that lack of insurance coverage is the primary reason for declining treatment [4]. Some patients report being bothered by the diagnosis of seborrheic keratosis, even when it is not symptomatic, and they are educated that it is not cancerous. Patients who present with seborrheic keratosis as a primary diagnosis may remain concerned about the possibility of skin malignancy, even when informed it is benign. Also, patients report being bothered by their appearance, with some avoiding clothing that would expose their seborrheic keratoses and using makeup or hairstyles to cover seborrheic keratoses. Many patients express disappointment that treatment for seborrheic keratosis is not covered by insurance.

While cosmetic procedures may enhance a patient's self-esteem and psychosocial functioning [5], removal of non-symptomatic seborrheic keratoses is typically not covered by third-party payers, and patients need to incur out-of-pocket expenses. This is reasonable, as covering all procedures patients may elect to have would certainly be a challenge for health insurers, who need to minimize expenses to continue to provide sustainable coverage. Also, cosmetic treatments may bear the risk of being futile or excessive treatments that are not in patients' long-term interests and may have side effects that do not improve overall appearance, quality of life, or health. As an example, shaving off seborrheic keratoses may lead to scars, wound infections, keloids, etc. Thus, it is critical to balance autonomy, beneficence, and non-maleficence in the approach to caring for asymptomatic seborrheic keratoses.

This leads to the important consideration of navigating how to respond when patients report symptoms of seborrheic keratoses that do not fit with the clinical picture. It is plausible that from previous encounters with dermatologists, as well as from exploring online medical literature or other sources, patients may have learned the conditions that qualify for insurance coverage of seborrheic keratoses treatment. In a bid to avoid the burden of out-of-pocket payments, they may opt to state that they are experiencing some of the symptoms. This places the health provider in the conundrum of having to assess the merits of these self-reported symptoms. If a patient says the lesion "itches", "hurts", or bothers them in some way, is that enough to bill the health insurance for treatment of the lesion? What if the patient has multiple lesions that they claim are symptomatic in such a way? Would it be ethical to bill the health insurance company for the treatment of these lesions if there are concerns that the patient may be malingering? How can the dermatologist assure that the patient is not malingering to avoid the cost of cosmetic treatment of seborrheic keratoses while maintaining a focus on patient care?

This showcases that there is sometimes a fine line between a cosmetic procedure and a medically necessary one, and this needs to be well-delineated in the context of self-reported symptoms of bothersome seborrheic keratoses. However, current literature does not provide much guidance on how to navigate this, and there is certainly room for more discussion on evidence-based approaches. It would be valuable for healthcare stakeholders to work collaboratively to develop viable strategies on how to navigate this and to provide pearls for practice.
